# Clinical and genetic characterization of ten Egyptian patients with Wolf–Hirschhorn syndrome and review of literature

**DOI:** 10.1002/mgg3.1546

**Published:** 2020-11-20

**Authors:** Mona K. Mekkawy, Alaa K. Kamel, Manal M. Thomas, Engy A. Ashaat, Maha S. Zaki, Ola M. Eid, Samira Ismail, Saida A. Hammad, Hisham Megahed, Heba ElAwady, Khaled M. Refaat, Shymaa Hussien, Nivine Helmy, Sally G. Abd Allah, Amal M. Mohamed, Mona O. El Ruby

**Affiliations:** ^1^ Human Cytogenetics Department National Research Centre Cairo Egypt; ^2^ Clinical Genetics Department National Research Centre Cairo Egypt; ^3^ Pediatric Department Faculty of Medicine Fayoum unIversity Fayoum Egypt

**Keywords:** cytogenomic analysis, *LETM1* genes, *WHSCR1*, *WHSCR2*, Wolf–Hirschhorn syndrome

## Abstract

**Background:**

Wolf–Hirschhorn syndrome (WHS) (OMIM 194190) is a multiple congenital anomalies/intellectual disability syndrome. It is caused by partial loss of genetic material from the distal portion of the short arm of chromosome.

**Methods:**

We studied the phenotype–genotype correlation.

**Results:**

We present the clinical manifestations and cytogenetic results of 10 unrelated Egyptian patients with 4p deletions. Karyotyping, FISH and MLPA was performed for screening for microdeletion syndromes. Array CGH was done for two patients. All patients exhibited the cardinal clinical manifestation of WHS. FISH proved deletion of the specific WHS locus in all patients. MLPA detected microdeletion of the specific locus in two patients with normal karyotypes, while array CGH, performed for two patients, has delineated the extent of the deleted segments and the involved genes. *LETM1*, the main candidate gene for the seizure phenotype, was found deleted in the two patients tested by array CGH; nevertheless, one of them did not manifest seizures. The study emphasized the previous.

**Conclusion:**

WHS is a contiguous gene syndrome resulting from hemizygosity of the terminal 2 Mb of 4p16.3 region. The Branchial fistula, detected in one of our patients is a new finding that, to our knowledge, was not reported.

## Graphical abstract

clinical, neurological, and molecular cytogenetic analysis of 10 Egyptian patients diagnosed with Wolf–Hirschhorn syndrome (WHS). Diagnosis was confirmed by genetic analysis through karyotype, FISH, MLPA, and array CGH with a new clinical finding which that, to our knowledge, was not reported before in WHS, extending the phenotypic spectrum of the disorder.

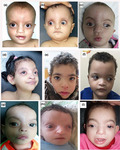

## 1 INTRODUCTION

The Wolf–Hirschhorn syndrome (WHS) (*OMIM 194190*) is a multiple congenital anomalies/intellectual disability syndrome, which affects 1 in 20,000 to 1 in 50,000 live births with a 2:1 female‐to‐male ratio (Maas et al., 2008). It was firstly described by Cooper and Hirschhorn (1961) and then by Hirschhorn et al. (1965) and Wolf et al. (1965).

WHS is associated with a wide range of clinical features. The core phenotype include typical facial appearance called Greek warrior helmet facies (hypertelorism, short and broad nose, short philtrum, downturned mouth, and low set dysplastic ears), intellectual disability ranging from mild to severe, growth delay, hypotonia, seizures, and microcephaly (Cammarata‐Scalisi et al., 2019; Zollino, et al., 2008). Other manifestations include congenital heart defect, cleft palate, hearing loss, kidney and genito‐urinary tract malformations as hypospadias. Ophthalmological and dental abnormalities, as well as skeletal abnormalities like talipes, mesomelia, radioulnar synostosis, fused vertebrae and ribs, and hip dislocation can also be features of the syndrome. The syndrome is frequently described as a Greek warrior helmet facies (broad forehead with prominent glabella and shallow orbital arches) (Antonius et al., 2008; Malvestiti et al., 2013).

WHS is caused by a partial loss of genetic material from the distal portion of the short (p) arm of chromosome 4 (4p16.3) which has a variable size that reflects the spectrum and severity of the disease. It is considered as a contiguous gene syndrome (Battaglia et al., 2008, 2015; Zollino, et al., 2008). Approximately 120 cases were described using conventional karyotyping. WHS is diagnosed by karyotype and/or FISH. Submicroscopic deletions associated with this disorder have been more recently identified by multiplex ligation‐dependant probe amplification (MLPA) and array‐CGH (Ho et al., 2016; Wright et al., 1997). Most cases are due to *de novo* deletion of chromosome 4p, while in 20% of cases; unbalanced translocation in the parental karyotype that includes chromosome 4 can cause the deletion (Battaglia et al., 2009). Complex genomic rearrangements as ring 4 chromosome may also be present in a smaller number of cases (Battaglia et al., 2015; Luo et al., 2011). Consequently, deletions detected in WHS vary in size and genetic content, resulting in the variability in presentation of this disorder. In more than 95% of cases, deletions are diagnosed by fluorescent *in situ* hybridization (FISH) using Wolf–Hirschhorn syndrome critical region (WHSCR) (*OMIM 194190*) specific probes (Bergemann et al., 2005; Zollino et al., 2004). Deletions in the *WHSCR* region are considered as the hallmark of WHS. Mapping efforts have identified two different sized overlapping deletions defining the WHS critical region 1 and 2 (*WHSCR1 and 2*) (South et al., 2007). These regions are suggested as being responsible for at least two of the core clinical manifestations of WHS, the developmental delay and the facial gestalt (Izumi et al., 2010). Microarray use to characterize chromosomal rearrangements led to many studies aiming at detecting genotype–phenotype correlation in WHS, and many of these studies described the regions of susceptibility to seizures and microcephaly in patients with WHS (Concolino et al., 2007; Ho et al., 2016; Maas et al., 2008; Shimizu et al., 2014; Zollino, et al., 2008).

The present work aims at characterizing the different clinical manifestations and cytogenetic abnormalities with genotype/phenotype correlation in 10 Egyptian patients with WHS.

## 2 SUBJECTS AND METHODS

### 2.1 Clinical evaluation

This study included 10 patients from 10 unrelated Egyptian families. All cases were subjected to full history taking including personal, medical, and developmental history with pedigree construction and analysis with special emphasis on parental consanguinity and other similarly affected family members. Complete general examination was carried out and essential anthropometric measures were performed including height, weight and head circumference according to the international biological program (Tanner et al., 1969). Investigations including brain MRI, EEG, Echocardiography, hearing test, and abdominal ultrasound were performed whenever indicated.

This study was approved by the Ethical Scientific Committee of the National Research Centre (NRC), Cairo, Egypt and was conducted in accordance with NRC by laws for human research. It conforms to the provisions of the Declaration of Helsinki in 2000. This study was conducted in the Clinical Genetics Department and the Human Cytogenetics Department, National Research Centre.

### 2.2 Cytogenomic analysis

#### 2.2.1 Conventional Giemsa Trypsin banding (GTG banding)

Chromosomal preparations were performed from peripheral blood samples collected in lithium heparin vacutainers, following standard protocols. Metaphase spreads were prepared and banded using GTG‐banding technique at an approximately 550 band level (Verma & Babu, 1995).

Karyotype description followed the recommendations of the International System for Human Cytogenomic Nomenclature (ISCN, 2016).

#### 2.2.2 Fluorescence in situ hybridization (FISH)

FISH was carried out using Cytocell commercial probes according to the manufacturer instructions (Cytocell FISH probes). The Wolf–Hirschhorn locus specific probe was used in all cases. It covers the Wolf–Hirschhorn critical region and extends between D4S168 and D4S43 (spectrum red) with a control region at 4qter (spectrum green). 4p and 4q subtelomere probes (spectrum green and spectrum red, respectively) were used in the two ring 4 patients. The procedure was done according to the manufacturer's instructions. Hybridizations were analyzed using Zeiss Axio Plan Microscope (Zeiss). Images acquisitions were performed using a CCD camera and analyzed using the ISIS program (In Situ Imaging System) (MetaSystems).

#### 2.2.3 Multiplex Ligation Dependent Probe Amplification (MLPA)

DNA was extracted from 5 ml of venous blood collected on PAXgene tube by PAXgene Blood DNA kit 761133 (PreAnalytix). The quantity and quality of DNA were assessed using a Nanoquant spectrophotometer and gel electrophoresis.

MLPA assay was performed for two patients (patients 2 and 3) using the SALSA MLPA Probemix P245‐B1 Microdeletion Syndromes‐1A, according to the manufacturer's instructions (MRC‐Holland). Separation of amplified products was done using Genetic Analyzer ABI 3500. Interpretation of the results was done utilizing Coffalyser. Net software (MRC‐Holland). Ratios less than 0.75 were considered as deletion, between 0.75 and 1.30 as normal and more than 1.30 as duplication.

#### 2.2.4 Array Comparative Genomic Hybridization analysis (array‐CGH)

DNA was extracted from 5 ml of venous blood similar to that described in MLPA analysis. Array‐CGH technique was performed for two patients (patient 1 and 7) to characterize the accurate size of deletion and the involved genes.

HD microchips were used, which are able to detect copy number variants (deletions, duplications) and single nucleotide polymorphism (SNP) (Affymetrix). In 4 days workflow, we performed DNA digestion, ligation, amplification, purification, fragmentation, labeling and then loading to the microchips and hybridization for 16 hours in Gene chip hybridization oven 645 (Affymetrix). After hybridization, the microchips were washed and stained in fluidic station 450 (Affymetrix). The microchips were scanned in Gene chip scanner 3000 (Affymetrix). Data analysis was done through chromosome analysis suit (CHAS) software.

## 3 RESULTS

### 3.1 Clinical data

**FIGURE 1:**
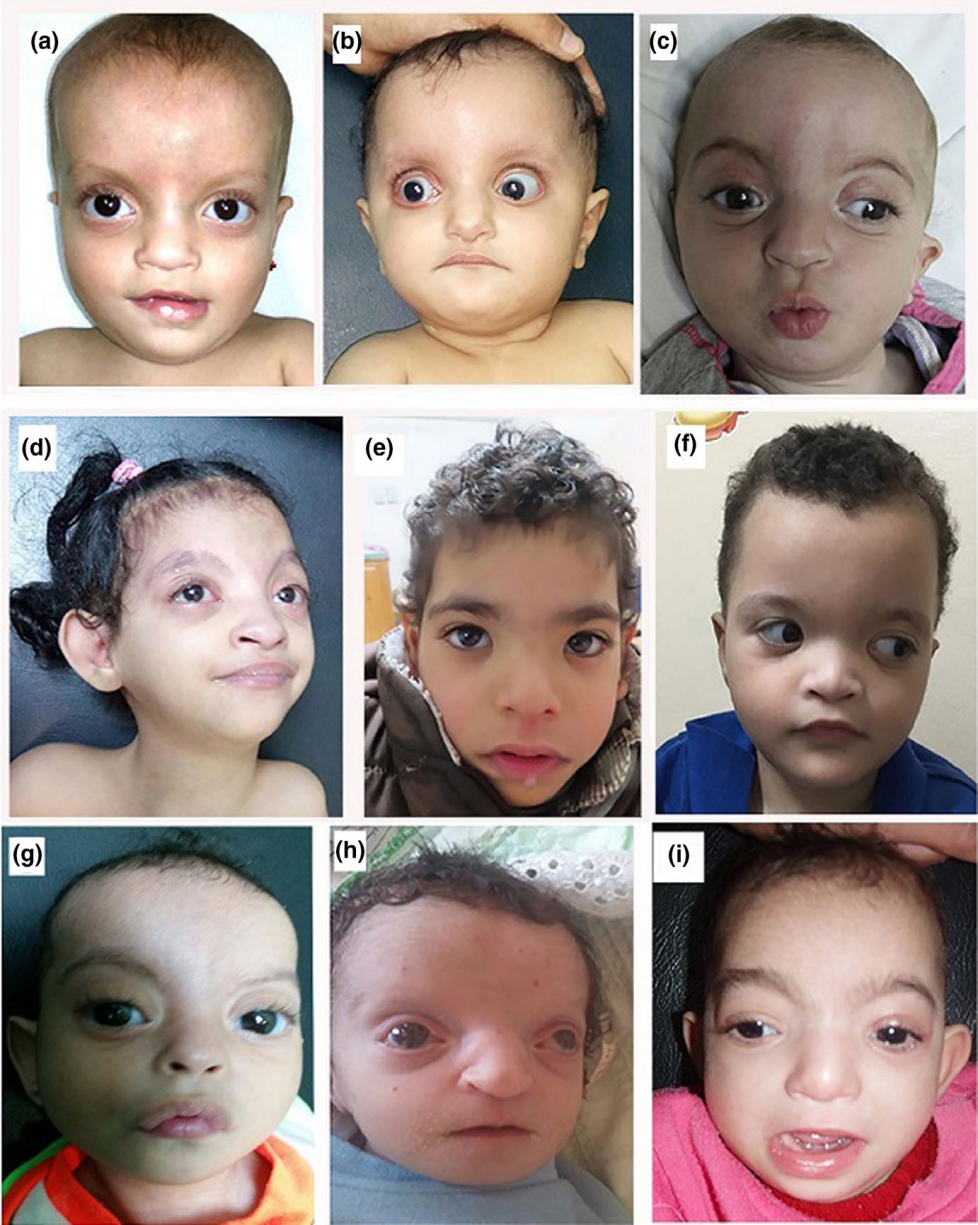
Facial features of the patients showing the Greek warrior helmet, frontal bossing, sparse scalp hair, low set ears, broad nasal bridge, hypertelorism, epicanthic folds, high forehead, proptosis and ectropion. Ptosis in pt. no. 8, 9 &amp; 10; squint in pt. no. 7 &amp; 9; upward eyelid slanting in pt. no. 1, 2, 5, 8 &amp; 10; downward eyelid slanting in pt. no. 3, 6, 7 &amp; 9

**FIGURE 2:**
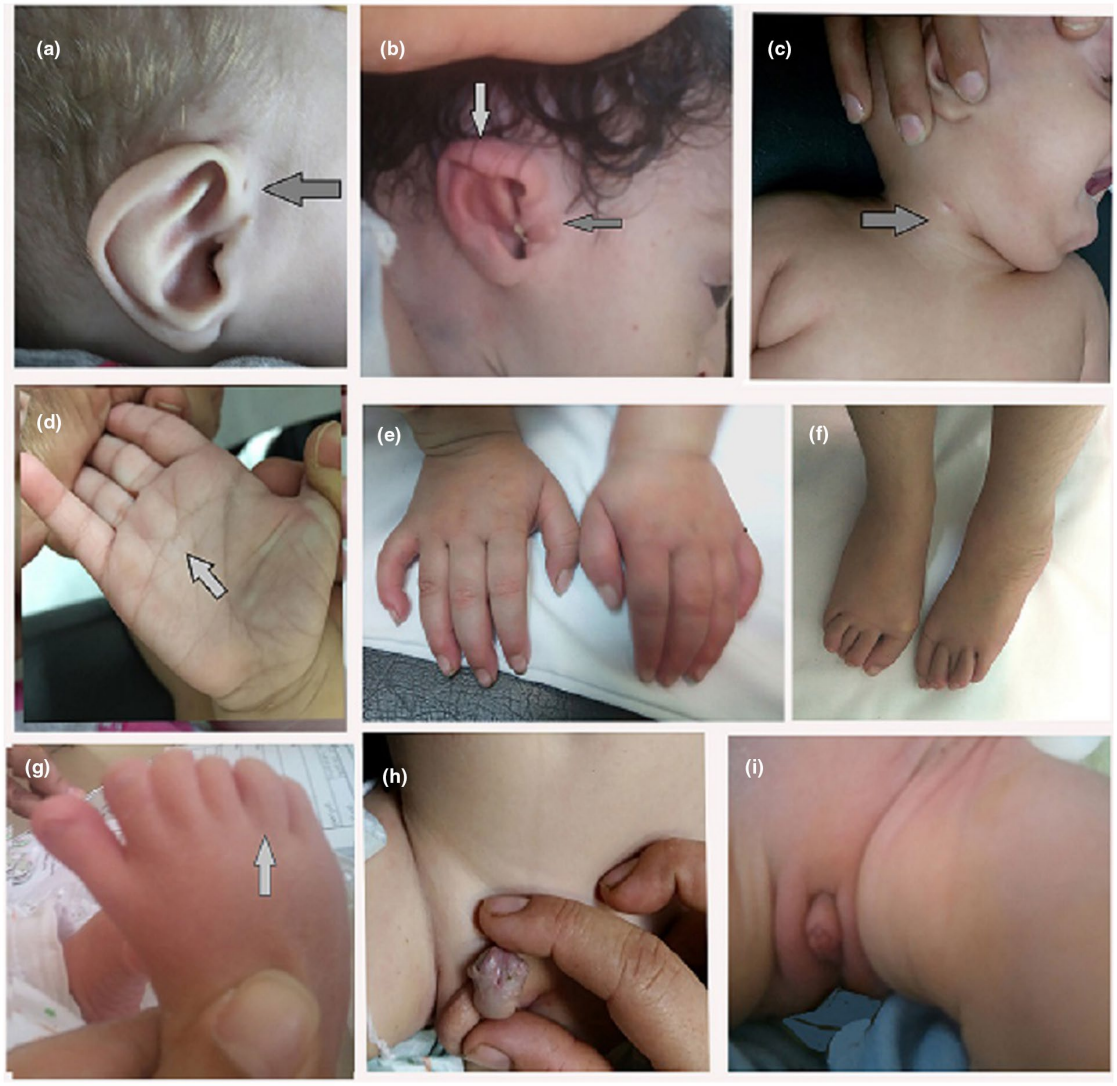
(a and b) preauricular tag, malformed ear, folded helix (pt. no. 3 &amp; 9). (c) branchial fistula (pt. no. 2). (d) ridged Simian crease (pt. no. 3 &amp; 7). (e) bilateral clinodactyly of the 5th finger (pt. no. 4). (f) overriding toes (pt. no. 4). (g) polydactyly of toes (pt. no. 9). (h) penile hypospadius (pt. no. 3 &amp; 8). (i) clitoromegaly (phallus like) (pt. no. 9)

**FIGURE 3:**
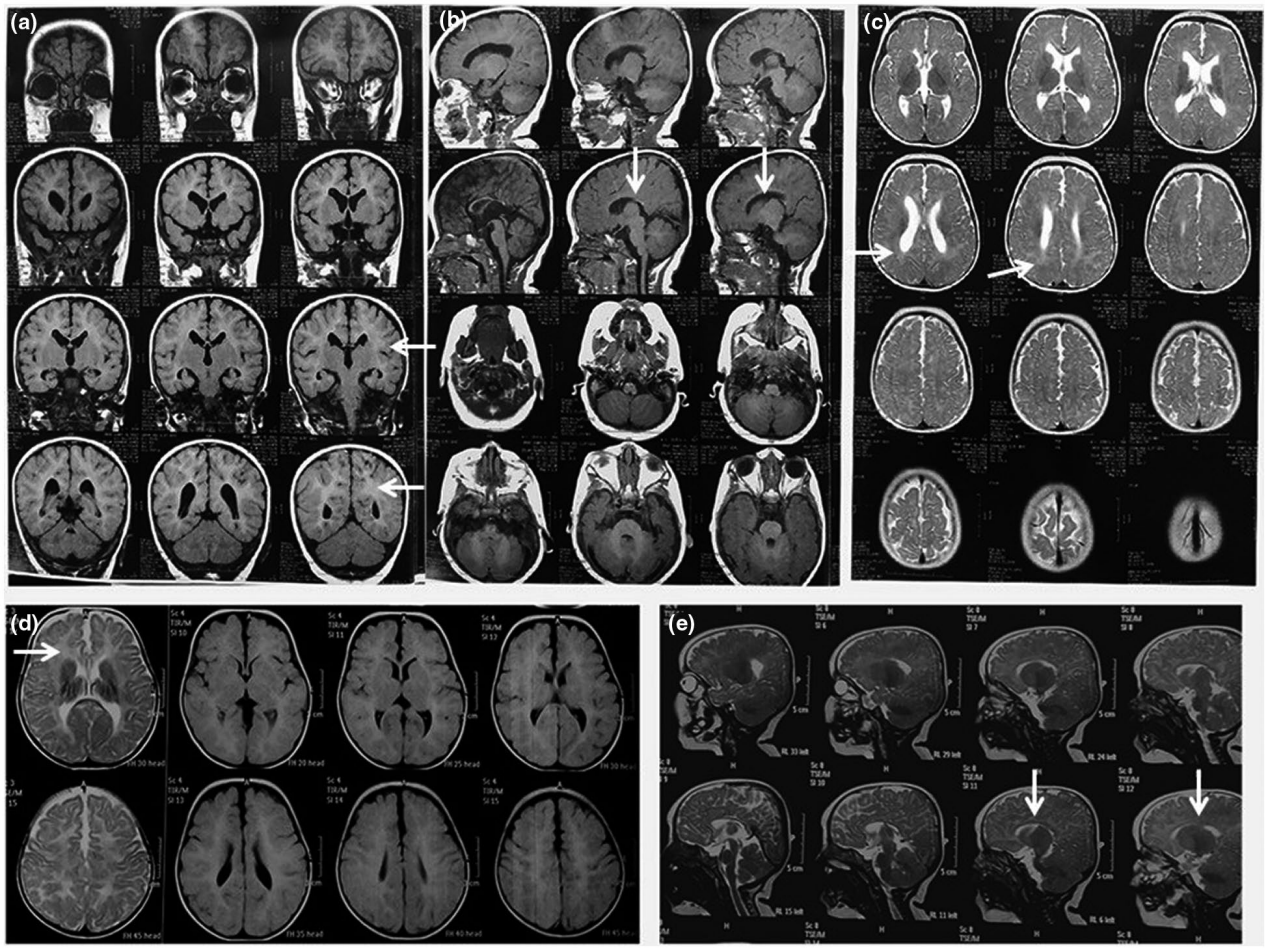
MRI brain showing: (a) Coronal T2‐FLAIR cut showing hypogenesis of corpus callosum and deep white matter dysmyelination (arrow) (pt. no. 7). (b) Saggital T1 showing thin corpus callosum (arrow), axial T1 showing normal posterior fossa (pt. no. 7). (c) Axial T2 showing mild dilatation of lateral ventricles which are separated apart indicating hypogenesis of corpus callosum and deep white matter defective myelination (arrow) (pt. no. 7). (d) Axial T2 and T2 FLAIR showing defective myelination (arrow) (pt. no. 1). (e) Saggital T2 showing thin corpus callosum (hypogenesis) (arrow) (pt. no. 1)

Our study included 10 patients, three males and seven females. Their ages ranged from 1/12 to 5 8/12 years. Parental consanguinity was present in four patients (40%). Four of the patients had a history of IUGR. Detailed clinical manifestations of our patients are summarized in Table 1. All patients had motor and cognitive delay, and all displayed growth delay, microcephaly with the characteristic facial features (the Greek warrior helmet fancies) indicative of WSH syndrome (Figure 1). Seizures occurred in 70% of the patients. Short stature and some skeletal changes in the form of talipes, hip dislocation, polydactyly of toes and overriding of toes were also observed (Figure 2). Hypotonia was evident in 8 patients, while patient no. 2 presented with hypertonia. Autistic features were observed in three patients (no. 5, 6, and 7). Eye manifestations including hypertelorism, epicanthic folds and ectropion were present in all patients. Ptosis (Pt. no.8, 9, &10), squint (Pt. no.7 & 9), optic nerve dysfunction (Pt. no.3, 4 & 8), retinal and macular dysfunction (Pt. no.3 & 8) were also observed. Other manifestations including cleft palate (Pt. no.1 & 2), bilateral branchial fistula (Pt. no 2) (Figure 2), diaphragmatic hernia (Pt. no. 3), and conductive hearing loss (Pt. no. 3) were also documented. Hypospadias was present in patients no. 3 and 8 and bilateral cryptorchidism was detected in patient no 3 (Figure 2). Fifty percent displayed congenital heart defects (no. 1, 2, 4, 8, & 9), including patent foramen ovale (PFO) in four patients and ASD in two patients (case no. 4 & 8). MRI of the brain showed agenesis or hypogenesis of the corpus callosum in all patients and cerebral white matter defective myelination was present in two patients (Pt. no. 1 & 7) (Figure 3).

**Table 1:** showing main clinical evaluation and investidations of the WSH patients

	Pt. 1	Pt. 2				Pt. 3			Pt. 4			Pt. 5				Pt. 6			Pt. 7			Pt. 8			Pt. 9				Pt. 10	
Age	9/12 years	8/12 years				1 years			5 years			5 8/12 years				3 9/12 years			1 9/12 years			11/12 years			1/12 years				2/12 years	
Sex	F	F				M			F			F				F			M			M			F				F	
Consanguinity	−	−				−			+			−				−			−			+			+				+	
Parental age																														
Mother	31 years	23 years				35 years			46 years			28 years				23 years			34 years			29 years			32 years				22 years	
Father	41 years	26 years				42 years			56 years			34 years				29 years			42 years			33 years			42 years				33 years	
Growth evaluation																														
Wt, SD	5 kg (−4 SD)	6 kg (−2.2 SD)				5 kg (−4.9 SD)			9.5 kg (−3.3 SD)			10 kg (−3.1 SD)				11 kg (−3.1 SD)			8 kg (−3 SD)			6.5 kg (−3.6 SD)			3 kg (−0.7 SD)				2.5 kg (−4.7 SD)	
Ht, SD	60 cm (−4 SD)	61 cm (−3.1 SD)				67 cm (3.3 SD)			87 cm (−4.5 SD)			90 cm (−3.8 SD)				89 cm (−2.6SD)			73 cm (−3 SD)			66 cm (−3.8 SD)			50 cm (+0.2 SD)				44 cm (−6.9 SD)	
HC, SD	40 cm (−3 SD)	40.5 cm (−2.5 SD)				39 cm (−4.3 SD)			40.5 cm (−7 SD)			43 cm (−6SD)				47.5 cm (−2.1 SD)			43.5 cm (−3.1 SD)			40 cm (−4.1 SD)			35 cm (+0.6 SD)				29.5 cm (−6.6 SD)	
IUGR	−	+				−			+			−				−			+			−			−				+	
Global developmental delay	+	+				+			+			+				+			+			+			+				+	
Facial dys‐morphism																														
Frontal bossing	+	+				−			+			+				+			+			+			+				−	
Brachycephaly	−	+				−			−			−				−			+			+			+				−	
Sparse scalp hair	+	+				+			+			+				+			+			+			−				−	
Proptosis	+	+				+			+			+				+			+			+			+				+	
Ptosis	−	−				−			−			−				−			−			+			+				+	
Ectropion	+	+				+			+			+				+			+			+			+				+	
Upslanted slanted palpebral fissures	+	+				−			−			+				−			−			+			−				+	
Downslanted palpebral fissures	−	−				+			−			−				+			+			−			+					
Loss of lower eye lid lashes	+	+				−			+			+				+			−			+			−				−	
Hypertelorism	+	+				−			+			+				+			−			+			+				−	
Epicanthic folds	−	−				−			+			−				−			−			+			−				−	
Low set ears	+	+				+, (bilat. Pre‐auricular tag)			+			+, (protru‐ded ears)				+			+			+, (large cupped)			+, (Rt preauricular tag, folded helix)				+	
"Greek warrior helmet appearance"	+	+				+			+			+				+			+			+			+				+	
Oral exam.																														
Tongue tie	+	+				−			−			+				−			−			+			−				−	
Micrognathia	−	+				+			−			−				+			−			+			+				+	
Short philtrum	+	+				−			+			+				+			−			−			+				−	
Tented upper lip	+	+				−			+			+				+			−			+			+				−	
Cleft palate	+	+				−			−			−				−			−			−			−				−	
CNS exam.																														
Tone	N	Hyper‐tonia				Hypo‐tonia			Hypo‐tonia			Hypo‐tonia				Hypo‐tonia			Hypo‐tonia			Hypo‐tonia			Hypo‐tonia				Hypo‐tonia	
Tendon Reflexes	Brisk	Brisk				Brisk			Hypo‐reflexia			Brisk				Brisk			N			Brisk			N				Hypo‐reflexia	
Autistic features	−	−				−			−			+				+			+			−			−				−	
Microcephaly	+	+				+			+			+				+			+			+			+				+	
Seizures/EEG changes	+/+	−/−				+/+			+/+			+/+				+/+			−/−			+/+			−/−				+/+	
External genitalia	N	N				Hypo‐spadius & bilat. Crypto‐rchidism			N			N				N			N			Penile hypo‐spadius			Clitoro‐megaly (phallus like)				N	
Eye examination																														
Squint				−				−		−			−		−			−			+			−			+			−
obstructed lacrimal duct				+				+		−			−		−			−			−			+			−			−
Hypermetropia				−				+		−			−		+			−			−			−			−			−
Optic N. dysfunction				−				−		+			+		−			−			−			+			−			−
Retinal & macular dysfunction				−				−		+			−		−			−			−			+			−			−
Other findings																														
Short stature			+		+		+				−			+			−			+			+			+		+		
Single transverse palmar crease			−		−		+				−			−			−			+			−			−		−		
Talipes			−		+		−				−			−			−			−			−			−		+		
Polydactyly of toes			−		−		−				−			−			−			−			−			+		−		
Overriding of toes			−		−		−				+			−			−			−			−			−		−		
Bilat. hip dislocation			−		−		+				+			−			−			−			−			−		−		
Bilateral branchial fistula			−		+		−				−			−			−			−			−			−		−		
Diaphragmtic hernia			−		−		+				−			−			−			−			−			−		−		
Intellectual disability			+		+		+				+			+			+			+			+			+		Cannot be assessed		
Hearing loss			−		−		+, (Conduc‐tive)				−			−			−			−			−			−		Cannot be assessed		
ECHO																														
PFO			+		+		−				−			−			−			−			+			+		−		
ASD			−		−		−				+			−			−			−			+			−		−		
PHT			+		−		−				−			−			−			−			−			−		−		
MRI																														
Agenesis of CC			+		+		+				+			Hypo‐genesis of CC			Thin CC			Thin CC			Thin CC			+		+		
White matter degeneration			+		−		−				−			−			−			+			−			−		−		

Abbreviations: ASD, atrial septal defect; CC, corpus callosum; exam, Examination; HC, head circumference; Ht, height; IUGR, intrauterine growth retardation; N, normal; PFO, Patent foramen oval; PHT, pulmonary hypertension; Pt, patient; SD, standard deviation; Wt, weight.

### 3.2 Cytogenomic data

**FIGURE 4:**
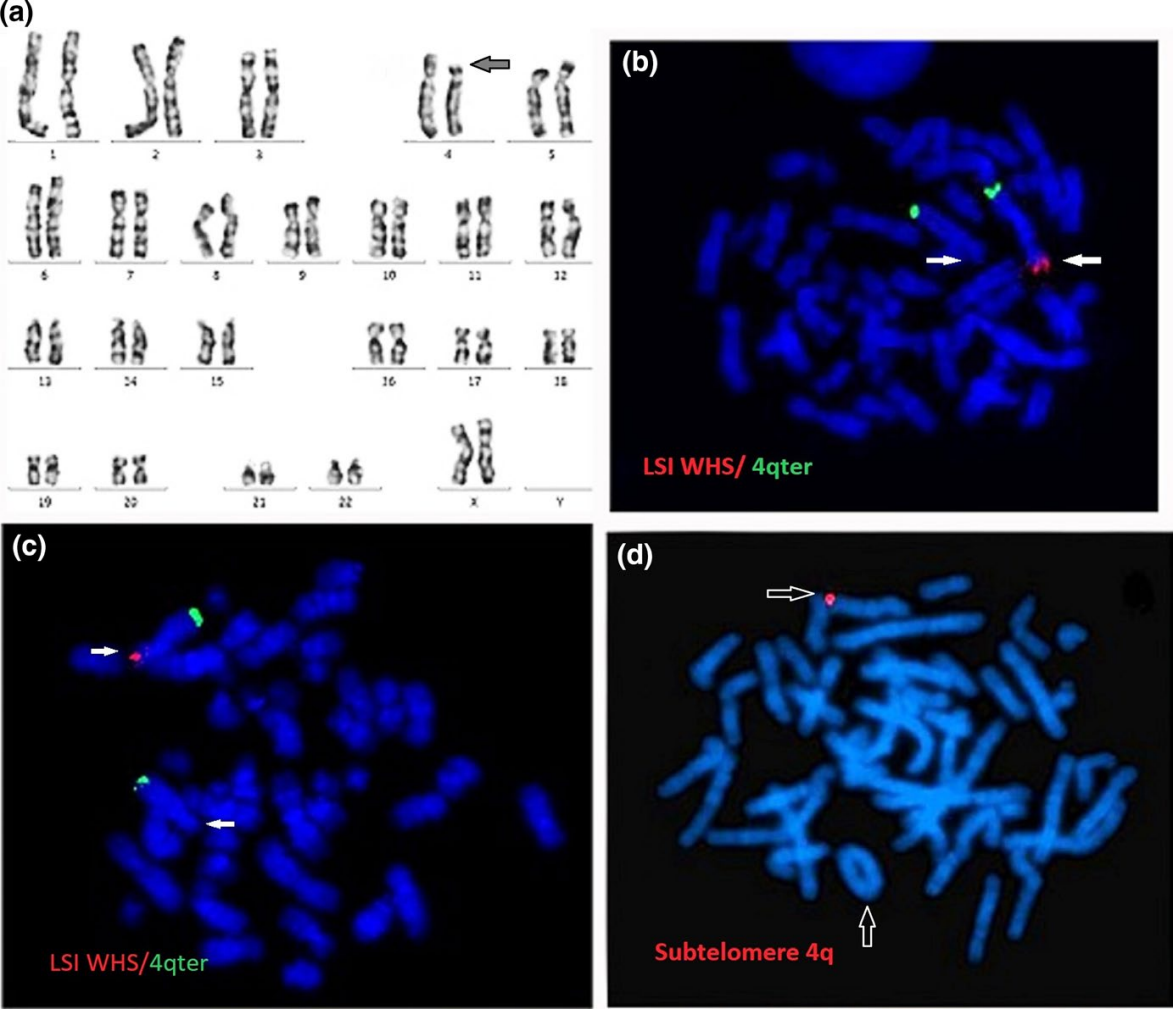
FISH (pt. 9 &amp; 10): (a) A GTG banded karyotype showing del(4)(p16.2). (b and c) FISH on a blood metaphase of patients 1 and 2 using LSI WHS probe (red) with 4qter (green) showing only one red signal on one copy of chromosome 4 with deletion in the other copy. (d) FISH on a blood metaphase of patient 10 using 4q subtelomere (red) showing deletion of this region on the ring 4 (arrow)

**FIGURE 5:**
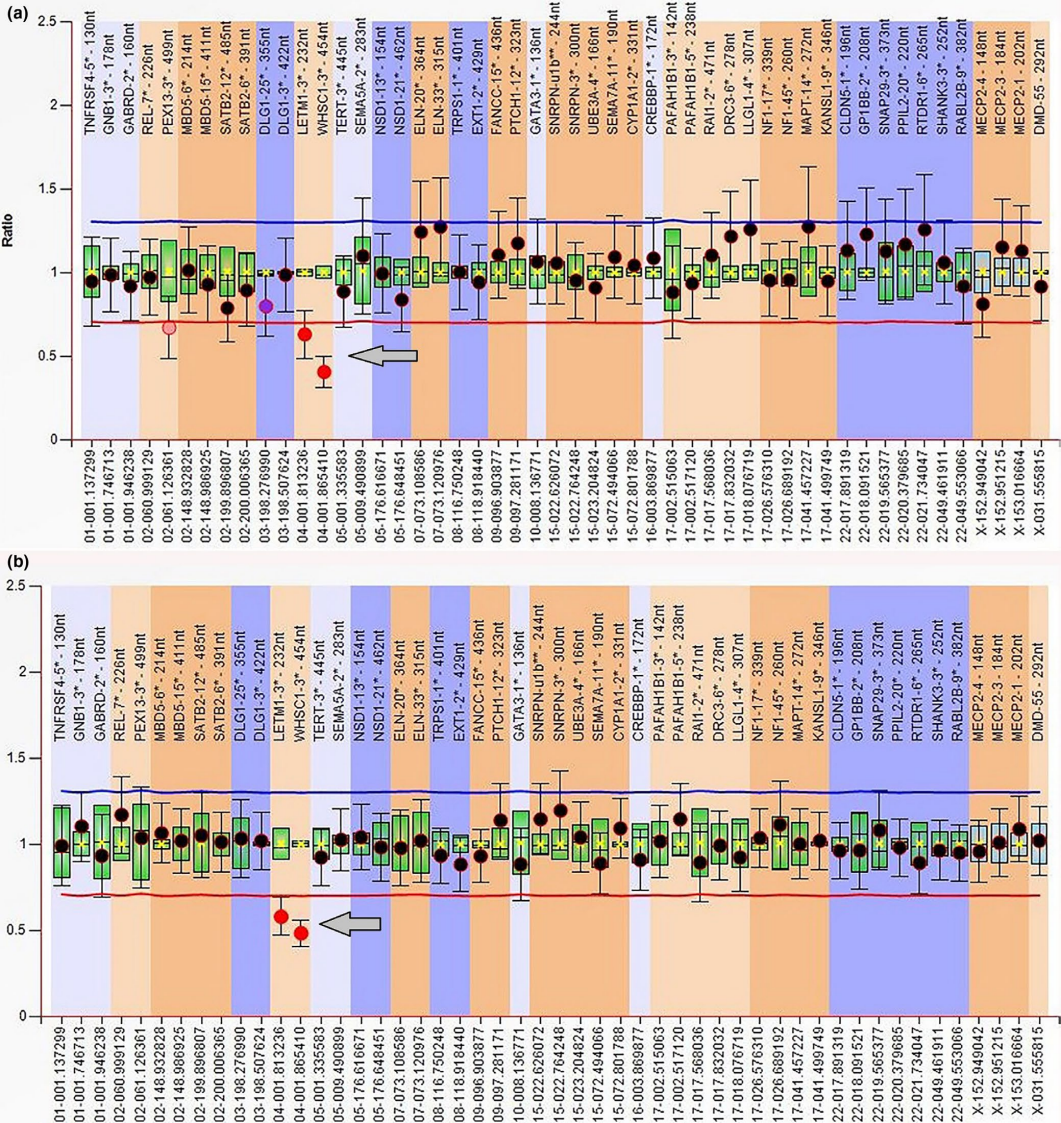
(a and b) MLPA (pt. no. 2 &amp; 3): Ratio chart interpretations of the data by the Coffalyser software showing a heterozygous deletion in WHS region in patients 2 and 3, respectively, indicated by a ratio tending toward 0.5 (arrow), (ratio of 0 would indicate homozygous deletion)

**FIGURE 6:**
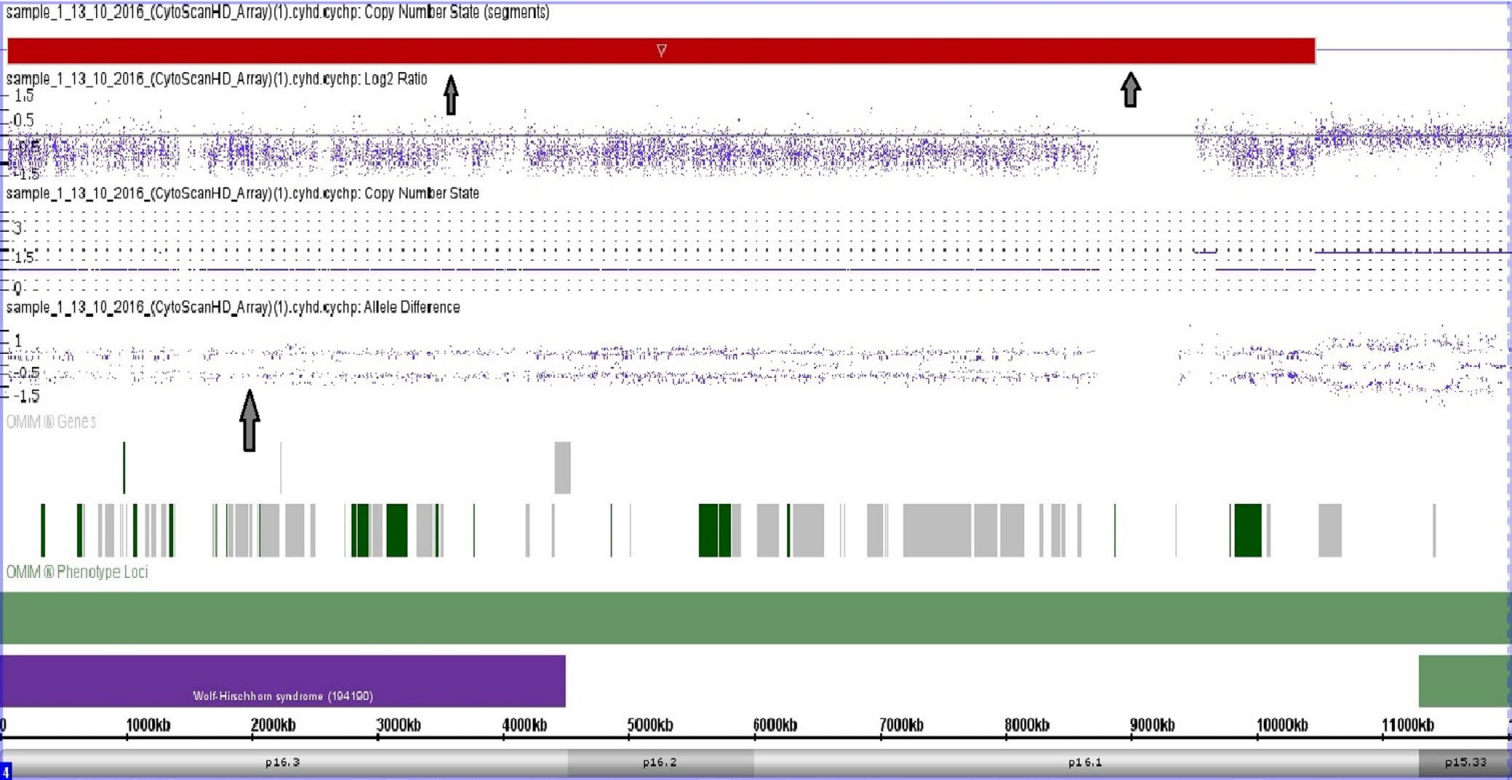
array‐CGH (Pt. no. 1): Use of the ChAS software tool with an Affymetrix HD microarray demonstrating a deletion in chromosome 4p. The log2 ratio is equal to 0.5, the CN state is equal to one; and there are only two allele difference tracs indicating terminal deletion of 10.4 Mb, arr[hg19]4p16.3p16.1(68,345_10,465,994)x1

Karyotype analysis revealed terminal deletions in six patients (Pt. no. 1, and 4 to 8) and a ring chromosome 4 in two patients (Patients 9 and 10) (Table 2; Figure 4). FISH analysis using locus specific probe for WHS revealed one hybridization signal, with two hybridization signals of the control region, indicating the deletion of WHS specific region on one copy of chromosome 4 in all patients. The use of 4p and 4q subtelomere probes revealed the deletion of both 4p and 4q subtelomeres in the ring chromosome 4 in patients 9 and 10 (Figure 4). Through screening for microdeletion syndromes by MLPA assay, two patients (2 and 3) showed submicroscopic deletion of WHS region, at 4p16.3 (Figure 5). The deletion was then confirmed by FISH analysis. Array‐CGH was performed for two patients (Pt. no. 1 & 7): case no. 1 showed terminal deletion of 10.4 Mb: arr[hg19] 4p16.3p16.1(68,345_10,465,994)x1 (Figure 6), while the other patient No.7 revealed 13.9 Mb terminal deletion: arr[hg19] 4p16.3p15.33 (71,552_13,977,366)x1.

**Table 2:** Results of Cytogenetic analysis including karyotyping, MLPA, FISH, and CMA

	Pt. 1	Pt. 2	Pt. 3	Pt. 4	Pt. 5	Pt. 6	Pt. 7	Pt. 8	Pt. 9	Pt. 10
Karyotype	46,XX,del(4)(p16.1)	46,XX	46,XY	46,XX, del(4)(p15.3)	46,XX, del(4)(p15.2)	46,XX, del(4)(p15.3)	46,XY, del(4)(p15.3)	46,XY, del(4)(p15.2)	46,XX,r(4)	46,XX,r(4)
MLPA	—	Del. WHS region (4p16.3)	Del. WHS region (4p16.3)	—	—	—	—	—	—	—
FISH	del(4)(p16.3)	del(4)(p16.3)	del(4)(p16.3)	del(4)(p16.3)	del(4)(p16.3)	del(4)(p16.3)	del(4)(p16.3)	del(4)(p16.3)	del(4)(p16.3).	del(4)(p16.3)
									Deletion of 4p and q subtelomeres	Deletion of 4p and q subtelomeres
CMA	arr[hg19] 4p16.3p16.1 (68,345_10,465,994)x1.	—	—	—	—	—	arr[hg19] 4p16.3p15.33 (71,552_13,977,366)x1.	—	—	—

Abbreviations: CMA, chromosomal microarray; FISH, fluorescence in situ hybridization; MLPA, multiplex ligation‐dependant probe amplification; Pt, patient; WHS, Wolf–Hirschhorn syndrome.

## 4 DISCUSSION

The Wolf–Hirschhorn syndrome (WHS) (*OMIM 194190*) is one of the most common deletion syndromes and is caused by deletion (complete or partial) of the short arm of chromosome 4. Herein we discuss the detailed clinical manifestations and cytogenetic analysis of 10 unrelated WHS Egyptian patients and compare our results with previously reported patients.

The frequency of WHS in this study was more prevalent in females (70%), which is consistent with all previous reports (Li et al., 2009; Yang et al., 2016; Zollino, et al., 2008). All patients exhibited the characteristic clinical features of WHS, including growth delay (IUGR or postnatal growth delay), distinctive facial features (Greek warrior helmet facial appearance), and intellectual disability (Ji et al., 2010; Yang et al., 2016; Zollino, et al., 2008; Zollino et al., 2014). Hypotonia was detected in 80%, while epilepsy or EEG anomalies occurred in 70% of patients, which is less than that reported in many previous studies to be 80%–90% (Battaglia et al., 2009; Wieczorek et al., 2000; Yang et al., 2016; Zollino, et al., 2008). Similar to our study, postnatal short stature and microcephaly, intellectual disability and hypotonia were reported in many studies with a frequency approaching100 percentage (Battaglia et al., 1999; Wieczorek et al., 2000; Zollino, et al., 2008). In most patients with WHS, the phenotype results from de novo chromosomal terminal deletions involving chromosome region 4p16.3. However, complex rearrangements, mainly unbalanced translocations may occur in some patients. We have detected eight classical terminal 4p deletions (in 80% of our patients), while two (Pt. no. 2 & 3) were diagnosed by MLPA analysis as a microdeletion not evident by conventional karyotyping (less than 5 Mb). Two patients had ring chromosome 4 (Pt. no 9 & 10). Further delineation of the deleted region was performed for patients No.1 and 7, using array‐CGH. While only two patients carried 4p microdeletion detected by MLPA in our study, Maas et al. (2008) reported 21 WHS patients with pure 4p deletions, 13 of them had submicroscopic deletions, reflecting the deletion size variability and emphasizing the importance of molecular cytogenetic technique for WHS detection. Zollino, et al. (2008) identified four different cytogenetic categories of chromosome 4 rearrangements in WHS patients: The major one was isolated 4p deletions, accounting for about 70% of patients. They were usually terminal, but interstitial deletions preserving the 4p subtelomere were also detected. Unbalanced translocations accounted for about 22% of cases and inverted duplications of 4p with terminal deletions occurred in 6%. Derivative chromosome 4 resulting from unbalanced pericentric inversion was detected in 2% of cases with a large duplicated 4q segment on the deleted 4p. Similarly, the majority of the 22 Japanese patients reported by Shimizu et al. (2014) had terminal 4p deletions (20 patients), and only two patients had interstitial deletion. A high prevalence of de novo t(4;8)(p16;p23) translocations was noticed in different large series of patients (South et al., 2008; Wieczorek et al., 2000; Zollino, et al., 2007; Zollino et al., 2004). They could be associated with a maternal inversion polymorphism on 4p16 or arise during maternal meiosis due to homologous non‐allelic recombination (NAHR) mediated by olfactory receptor gene clusters on both 4p and 8p (Maas et al., 2008). Buggenhout et al. (2004) suggested that low copy repeats not only mediate ectopic meiotic recombination but also susceptibility sites for terminal deletions. Zollino et al. (2007) investigated the parental origin of 73 WHS‐associated rearrangements and concluded that parental 4p16.3 inversion polymorphism is not a risk factor for WHS‐associated rearrangements.

The severity of the clinical presentation has been correlated to the size of the deleted region and the breakpoint site (Ho et al., 2016; Shimizu et al., 2014; Yang et al., 2016; Zollino, et al., 2008). Three major phenotypic groups were defined as mild, moderate and severe. The first comprises a small deletion of 3.5 Mb or less and is more likely under diagnosed. The moderate second type is the more frequent category with deletions between 5 and 18 Mb and usually have the typical WHS features. The third severe category results from very large deletions of 22–25 Mb or more and is characterized by additional severe complex features, including typical facial appearance, severe intellectual disability, severe growth delay, severe seizures, neurological abnormalities, ophthalmic abnormalities, congenital heart malformations, skeletal, renal anomalies and cleft palate and hypospadias (Zollino, et al., 2008). Nonetheless, the cardinal phenotypic features of WHS are thought to result from contiguous gene regions, of which deletion is sufficient to result in WHS characteristic features. Many of these genes are yet to be identified, however, several candidate genes were proposed especially for the seizure phenotype (Ho et al., 2016). Comparative molecular analysis of terminal and interstitial deletions enabled identification of the first WHS critical region, which was the shortest deletions overlap region (SRO) that was confined to a 165 kb, about 2 Mb from the telomere (Wright et al., 1997). Two main candidate developmental genes were identified within this region, the first was designated WHS candidate‐1 (*WHSC1*; Stec et al., 1998) and the second was Wolf‐Hirschhorn syndrome candidate‐2, (*WHSC2*; Wright et al., 1999). Nimura et al. (2009) proposed that *WHSC1* functions as a transcriptional regulator together with developmental transcription factors to prevent various pathophysiologies. Deletion of *WHSC1*, abbreviated later as *NSD2* gene (*OMIM 602952*), can disrupt the regulation of several other genes resulting in WHS features including intellectual disability, growth delay, and a distinctive facial appearance (Jiang et al., ). De novo Loss of function mutations in *NSD2* gene were recently reported in patients with atypical WHS and in developmental delay, congenital cardiac defects and autism (Boczek et al., 2018; Jiang et al., ). *WHSC2* gene (now abbreviated as *NELFA* gene) (OMIM 606026) functions in histone mRNA maturation by linking with histone gene loci, cleavage bodies, and Cajal bodies in a temporally and spatially coordinated manners (Narita et al., 2007). Zollino et al., (2003) re‐defined a critical region, referred to as *WHSCR*‐*2*, within a 300–600 kb interval (at 1.6–1.3 Mb from the telomere). The region was contiguous, distally, to the previous one and shares with it the *WHSC1* gene. *LETM1* (*OMIM 604407*) is another candidate gene which maps to *WHSCR2*. It is involved in Ca2 signaling and is considered as the major candidate gene for seizure phenotype (Corrêa et al., 2018; Endele et al., 1999; Okamoto et al., 2013; Rauch et al., 2001; South et al., 2007; Yang et al., 2016). Many patients were reported without seizures and with interstitial deletions encompassing *LETM1* and preserving the distal region (Ho et al., 2016; Maas et al., 2008; Shimizu et al., 2014). Others had seizures associated with small distal 4p deletions not including *LETM1* (Faravelli et al., 2007; Ho et al., 2016; Maas et al., 2008; Misceo et al., 2012; Zollino, et al., 2008). Many authors suggested that the more distal region of 4p to be a candidate region for seizure phenotype (Ho et al., 2016; Misceo et al., 2012; Shimizu et al., 2014; South et al., 2008). Shimizu et al. (2014) suggested a small distal region including *CPLX1* and *CTBP1*genes. It was stated by Bi et al. (2016) that a synergism between *CPLX1*, *PIGG*, and *LETM1* genes was associated with seizures in patients with WHS. *CTBP1* gene (*OMIM 602618*) encodes a transcriptional regulator that interacts with chromatin‐modifying enzymes to regulate gene expression in many cellular pathways (Beck et al., 2016). The hemizygosity of *CTBP1* gene was described to be related to the progression of epilepsy in WHS patients (Simon & Bergemann, 2008). *CPLX1* (*OMIM 605032*) is a protein coding gene related to neurotransmitter release cycle and transmission across chemical synapses. Subsequently, Ho et al. (2016) refined the candidate seizure susceptibility region to a 197 kb locus starting 368 kb from the terminal 4p and encompassing two genes: *ZNF721* which is a protein coding gene of unknown function and *PIGG* (*OMIM 616918*), a member of the phosphatidylinositol glycan anchor biosynthetic pathway. Allelic variants of this gene have been associated with intellectual disability, hypotonia, and seizures of early onset (Makrythanasis et al., 2016). More recently, Corrêa et al., (2018) suggested a 330 kb region of susceptibility to convulsions and microcephaly in the terminal 4p16.3 encompassing seven genes *LETM1*, *FGFR3*, *WHSC1*, *NELFA*, *C4orf48*, *NAT8L*, *and POLN*. *C4orf48* gene encodes a short evolutionarily conserved protein. Endele et al. (2011) had proposed that *C4orf48* (*OMIM 614690*) may be involved in the intellectual and other neurological aspects of WHS syndrome. *NAT8L* (*OMIM 610647*) is a protein coding gene which has a role in regulation of lipogenesis by producing N‐acetylaspartate acid (NAA). This is a brain‐specific metabolite with a high concentration in brain and its hydrolysis play a significant role in maintaining an intact white matter (Martin et al., 2001). *POLN* gene (*OMIM*; *610887*) encodes a DNA polymerase type‐A family member, that plays a role in DNA repair and homologous recombination. *FGFR3* gene (*OMIM 134934*) encodes a member of the fibroblast growth factor receptor (FGFR) family, with a highly conserved amino acid sequence that plays an essential role in the regulation of cell proliferation, differentiation, and apoptosis, and is required for normal skeletal development. (Table 3 shows a list of all proposed candidate genes of WHS phenotype).

**Table 3:** Proposed genes for WHS characteristic features

Location (from NCBI, GRCh38)	Gene/locus	Gene name	Gene MIM number	Phenotype	Gene function	Inheritance	References
4:784,956 4p16.3	CPLX1, CPX1, EIEE63	Complexin 1	605032	Epileptic encephalopathy, early infantile, 63	A protein coding gene related to neurotransmitter release cycle and transmission across synapses	AR	McMahon et al. (1995), Reim et al. (2001)
4:1,211,443 4p16.3	CTBP1, HADDTS	C‐terminal binding protein 1	602618	Hypotonia, ataxia, developmental delay, and tooth enamel defect syndrome Progression of epilepsy in WHS patients	Encodes a transcriptional regulator that interacts with chromatin‐modifying enzymes to modulate gene expression in multiple cellular pathways	AD	Simon and Bergemann (2008), Beck et al. (2016)
4:499,199 4p16.3	PIGG, GPI7, MRT53	Phosphatidylinositol glycan anchor biosynthesis class G protein	616918	Mental retardation, autosomal recessive, 53 Intellectual disability, hypotonia and seizures of early onset	A member of the phosphatidylinositol glycan anchor biosynthetic pathway	AR	Makrythanasis et al. (2016)
4:1,793,292 4p16.3	FGFR3, ACH	Fibroblast growth factor receptor‐3	134934	Achondroplasia Craniosynostosis, skeletal dysplasia	Encodes a member of the fibroblast growth factor receptor (FGFR) family, with a highly conserved amino acid sequence	AD	Corrêa et al. (2018)
4:1,811,478 4p16.3	LETM1	Leucine zipper/EF‐hand‐containing transmembrane protein 1	604407	Considered as the major candidate gene for seizure phenotype in WHS	Involved in Ca2 signaling	AD	Endele et al. (1999), Dimmer et al. (2008)
4:1,871,392 4p16.3	NSD2, WHSC1, MMSET	Nuclear receptor‐binding SET domain protein 2	602952	WHS features including intellectual disability, growth delay and a distinctive facial appearance	Developmental transcription	—	Stec et al. (1998), Nimura et al. (2009)
4:1,982,716 4p16.3	NELFA, WHSC2	Negative elongation factor complex member A	606026	One of the contagious genes of WHS	Histone mRNA maturation	—	Wright et al. (1999), Narita et al. (2007)
4:2,035,609 4p16.3	C4orf48	Chromosome 4 open reading frame 48	614690	Involved in the intellectual and other neurological aspects of Wolf‐Hirschhorn syndrome	Exclusively expressed in different zones of brain tissue during cortical and cerebellar development	—	Endele et al. (2011), Corrêa et al. (2018)
4:2,059,326 4p16.3	NAT8L, CML3, NACED	N‐acetyltransferase 8‐like	610647	?N‐acetylaspartate deficiency Microcephaly developmental delay, seizures, and secondary microcephaly	Regulation of lipogenesis. Plays a significant role in the maintaining an intact white matter	—	Martin et al. (2001)
4:2,071,917 4p16.3	POLN	Polymerase, DNA, nu	610887	One of the candidate genes of WHS	Encodes a DNA polymerase type‐A family member	—	Corrêa et al. (2018)

Abbreviations: AD, autosomal dominant; AR, autosomal recessive; WHS, Wolf Hirschhorn syndrome.

Our 10 patients had different deletion sizes encompassing the critical WHS regions containing all the proposed genes. Three of our patients despite of their young ages, did not exhibit seizures or EEG abnormalities. One of those patients (Pt. 7), who was about to finish his second year of life, had a 10 Mb terminal deletion. The other two patients (Pt. 2) with 4p microdeletion and (Pt. 9) with a ring chromosome 4, were near the end of their first year of life (Table 1). Generally, seizures begin in WHS patients during the first 3 years of life, especially around 6–12 months of age (Battaglia et al., 2009). This highlights the complexity of the development of the seizure phenotype and lack of complete understanding of the responsible genes.

Ring chromosomes are rare structural abnormalities, usually resulting because of breakage of the distal segments of the short and long arms, with subsequent end joining. Almost all reported ring chromosome 4 patients had both prenatal and postnatal growth retardation and microcephaly, developmental delay and intellectual disability (Balci et al., 2006; Concolino et al., 2007; Paththinige et al., 2016). Both of our ring chromosome female patients (patients 9 and 10) had dysmorphic features with the characteristic facial appearance and a global developmental delay. Patient 10 also had intrauterine growth retardation, convulsion and severe microcephaly, while patient 9 did not exhibit intrauterine growth retardation, convulsions or EEG changes. Concolino et al. (2007) also reported a ring chromosome 4 patient not experiencing seizures. The clinical manifestations in our two patients with ring 4 are typical of WHS but with more severe clinical manifestations including eye malformations. It is thought that clinical phenotypes observed in patients with ring chromosome 4 are in part due to terminal deletion of both chromosome arms, in addition to the ring instability during mitotic divisions. Nonetheless, 4q terminal deletions may have a minor or no impact on the phenotype and may be inherited from a normal parent as reported by (Buggenhout et al., 2004; Descartes et al., 1996; Vona et al., 2014), and in an Egyptian family previously studied by our group with a terminal 4q35.1q35.2 deletion (unpublished data).

Consistent with similar studies, additional malformations, including cleft palate, ptosis, squint, hypertelorism, diaphragmatic hernia, cerebral, cardiac, genital and skeletal abnormalities were also observed in our study (Aquino et al., 2015; Dellavia et al., 2011; Paradowska‐Stolarz, 2014; Sukarova‐Angelovska et al., 2014). A branchial fistula was found in one patient (pt 2), a finding that, to our knowledge was not reported before in WHS. Wieczorek et al., (2000) reported congenital heart defects, cleft lip/palate and preauricular tags in WHS cases with a rate of 31%, 15%, and 15%, respectively. They speculated a correlation between these clinical features and the deletion size, as they detected cleft lip/palate and preauricular tags in cases with deletions more than 9 Mb, and CHD in patients with deletions more than 16 Mb. However, our study reported a preauricular tag in two patients, one with a microdeletion (Pt. 3), and the other with ring 4 (Pt. 9). Cleft palate was detected in two patients (Pt.1 and Pt. 2), with terminal 10.4 Mb deletion and a microdeletion, respectively. Maas et al. (2008) had also refined the candidate region of cleft lip/palate to lie between 0.3 and 2.3 Mb.

CHD affected five of our patients (50%) (No. 1,2,4,8, and 9), four of them had pure terminal 4p deletion including one case with microdeletion, and the fifth case had ring (4). Similarly, Maas et al. (2008) reported CHD in four of the eight patients having deletions of 3.7–14.8 Mb. They refined CHD region proximal from 3.7 Mb. Other patients with CHD and microdeletions less than 3.5 Mb had been also reported (Zollino, et al., 2008). Several other reports described different congenital cardiac abnormalities with a rate ranging from 33.3% to 68% (Battaglia et al., 1999; Tautz et al., 2010; Verbrugge et al., 2009; Wieczorek et al., 2000; Zollino et al., 2000). Nimura et al. (2009) assumed that *NSD2* gene might play a role in modulating the cardiac transcriptional network through collaboration with *NKX2.5* gene (a homeodomain transcription factor essential for cardiac development) to suppress their target genes (Bruneau, 2008).

WHS patients are commonly having structural cerebral anomalies (80%–100%), mostly in the form of reduction of cerebral white matter, dilatation of the lateral ventricles, and corpus callosum abnormalities (Righini et al., 2007; Battaglia et al., 2008). Our study showed abnormalities of the corpus callosum in all patients and cerebral white matter degeneration was detected in two patients (Pt.1 and Pt. 7).

Skeletal anomalies were detected in about 50% of our patients, a rate nearly similar to that reported in the literature (about 35%–60%) (Battaglia et al., 2008; Zollino, et al., 2008). Similar to previous reports, multiple ophthalmological abnormalities were reported in our patients in the form of hypertelorism, epicanthal folds, ptosis, downward eyelid slanting, squint, hypermetropia, optic nerve dysfunction, and retinal and macular abnormalities. However, proptosis was a common feature in all cases. Previous studies suggested a relationship between the severity of the ocular manifestations and the deletion size (Dickmann et al., 2009; Wu‐Chen et al., 2004). This observation, however, was not recorded in the present study.

Three of our patients (30%) (3, 8, 9) showed genital anomalies, one of them had hypospadius and bilateral cryptorchidism (Pt 3 with microdeletion), one had penile hypospadias (Pt. 8), and the third had clitoromegaly (pt. 9 with ring 4). Genital anomalies were previously reported with an average rate of about 45% (Wieczorek et al., 2000; Zollino, et al., 2008) and the critical region for hypospadias was mapped by Estabrooks et al. (1995) and Maas et al. (2008) to lie between D4S127 (3.0 Mb) and D4S10 (4.0 Mb).

The variability of clinical severity in our patients was not dependent on the size of the deleted segment, but rather on the involvement of the specific WHS candidate regions, empathizing that the deletion of contiguous WHS candidate genes is the cause of the characteristic clinical features of this syndrome. Similar observations were reported by Estabrooks et al. (1994, 1995) and Andersen et al. (2014). Variable severities observed in WHS patients depend on other factors as complex chromosomal abnormalities, associated submicroscopic CNV, gene penetrance, and epigenetic factors. Milder phenotypes however were observed in patients with microdeletions less than 3.5 Mb, who commonly skip diagnosis, whereas severe complicated phenotypes were detected in very large deletions of 22–25 Mb or more (Zollino, et al., 2008).

## 5 CONCLUSION

WHS is a contiguous gene syndrome resulting from hemizygosity of the 4p16.3 region. *WHSC1* (*NSD2*) gene is the main transcriptional regulator of several other genes involved in WHS phenotype. *LETM1* is an important seizure susceptibility gene; yet, other genes responsible for the seizure phenotype are present in the most distal terminal region, which is deleted in all of our patients. Branchial fistula, detected in one of our patients is a new finding that, to our knowledge, not reported before in WHS, extending the phenotypic spectrum of the disorder. MLPA is helpful as a screening strategy for patients clinically suspected to have WHS and can identify microdeletions that may not be detected by karyotyping. When available, higher throughput techniques as array‐CGH will be essential for accurate detection of the size of deleted segments and the candidate genes involved. These technique approaches can thus provide more precised understanding of the role and effect of genes involved in the causation of the syndrome. These can help in making an accurate genotype–phenotype correlation, correct diagnosis, establishing, providing a better patient management and also an appropriate genetic counseling.

## CONFLICT OF INTEREST

All authors declare no conflict of interest.
